# The transcription factor STAT5 drives mutation and imatinib resistance in chronic myeloid leukemia via ROS production

**DOI:** 10.1186/1471-2210-11-S2-A22

**Published:** 2011-09-05

**Authors:** Eva Grundschober, Wolfgang Warsch, Veronika Sexl

**Affiliations:** 1 Institute of Pharmacology and Toxicology, Veterinary University of Vienna, 1210 Vienna, Austria

## Background

Chronic myelogenous leukemia (CML) is a leukemic stem cell (LSC)-driven myeloproliferative disorder and is associated with a characteristic chromosomal translocation which generates a constitutively active tyrosine kinase, the BCR-ABL oncoprotein. The standard treatment therapy for CML patients is the BCR-ABL tyrosine kinase inhibitor (TKI) imatinib. It is a life-long treatment due to the fact that LSCs are resistant to TKIs. Since its introduction, imatinib has improved the 5-year survival rate up to 90%. An emerging problem is resistance to imatinib, which is mainly caused by mutations inside the BCR-ABL kinase domain, and its increasing incidence during disease progression. It has been reported that BCR-ABL drives its own mutation via upregulation of reactive oxygen species (ROS) causing oxidative DNA damage. Among the several dozens of intensively characterized mediators of BCR-ABL action, the transcription factor STAT5 is among the few ones that is critical for leukemia initiation and maintenance and it has been shown that STAT5 becomes upregulated during disease progression.

## Methods and results

qPCR analysis of primary CML patient samples reveal a positive correlation of STAT5 mRNA levels and BCR-ABL mutations. Using BCR-ABL transformed murine cell lines retrovirally overexpressing STAT5A or STAT5B, we can show that STAT5 triggers ROS production leading to an increase in DNA double-strand breaks.

## Conclusions

We hypothesize that STAT5 is an important mediator of imatinib resistance in CML due to its ability to drive ROS production consequently leading to BCR-ABL mutations.

